# Swing-phase detection of locomotive mode transitions for smooth multi-functional robotic lower-limb prosthesis control

**DOI:** 10.3389/frobt.2024.1267072

**Published:** 2024-04-12

**Authors:** Md Rejwanul Haque, Md Rafi Islam, Edward Sazonov, Xiangrong Shen

**Affiliations:** ^1^ Human-Centered Bio-Robotics Lab, Department of Mechanical Engineering, The University of Alabama, Tuscaloosa, AL, United States; ^2^ Computer Laboratory of Ambient and Wearable Systems, Department of Electrical and Computer Engineering, The University of Alabama, Tuscaloosa, AL, United States

**Keywords:** intent recognition, prosthesis control, wearable device, dynamic time warping, robotic lower limb prosthesis

## Abstract

Robotic lower-limb prostheses, with their actively powered joints, may significantly improve amputee users’ mobility and enable them to obtain healthy-like gait in various modes of locomotion in daily life. However, timely recognition of the amputee users’ locomotive mode and mode transition still remains a major challenge in robotic lower-limb prosthesis control. In the paper, the authors present a new multi-dimensional dynamic time warping (mDTW)-based intent recognizer to provide high-accuracy recognition of the locomotion mode/mode transition sufficiently early in the swing phase, such that the prosthesis’ joint-level motion controller can operate in the correct locomotive mode and assist the user to complete the desired (and often power-demanding) motion in the stance phase. To support the intent recognizer development, the authors conducted a multi-modal gait data collection study to obtain the related sensor signal data in various modes of locomotion. The collected data were then segmented into individual cycles, generating the templates used in the mDTW classifier. Considering the large number of sensor signals available, we conducted feature selection to identify the most useful sensor signals as the input to the mDTW classifier. We also augmented the standard mDTW algorithm with a voting mechanism to make full use of the data generated from the multiple subjects. To validate the proposed intent recognizer, we characterized its performance using the data cumulated at different percentages of progression into the gait cycle (starting from the beginning of the swing phase). It was shown that the mDTW classifier was able to recognize three locomotive mode/mode transitions (walking, walking to stair climbing, and walking to stair descending) with 99.08% accuracy at 30% progression into the gait cycle, well before the stance phase starts. With its high performance, low computational load, and easy personalization (through individual template generation), the proposed mDTW intent recognizer may become a highly useful building block of a prosthesis control system to facilitate the robotic prostheses’ real-world use among lower-limb amputees.

## 1 Introduction

Around the world, millions of people are living with major lower limb losses due to various causes such as injury and disease (Ziegler-Graham et al., 2008). Traditionally, passive (i.e., non-powered) prosthetic devices were used to restore the lost limb and joint (e.g., knee and ankle) functions. Due to the passive prostheses’ inability to generate active mechanical power, their users typically suffer from multiple issues in gait, e.g., asymmetric gait, increased hip power, and elevated metabolic energy consumption (Waters et al., 1976; Hof et al., 2007; Winter, 2009). Further, amputees fitted with passive prostheses experience significant difficulty in energetically demanding locomotive activities such as stair climbing (Bae et al., 2009), causing major inconveniences in their daily life. Motivated by these significant issues, multiple robotic (powered) lower-limb prostheses were developed by researchers in academia and industry [e.g., Vanderbilt Leg (Lawson et al., 2014) and Open-Source Leg (Azocar et al., 2020)], providing the potential to significantly improve amputee users’ mobility and quality of life through actively powered prosthetic joints. With two commercial products in clinical use [Ossur Power Knee (Cutti et al., 2008)] and Otto Bock Empower Ankle the capability of powered prostheses in restoring healthy-like gait in walking has been demonstrated in multiple studies (Johansson et al., 2005; Silver-Thorn and Glaister, 2009).

With their actively powered joints, robotic prostheses can potentially function like healthy biological limbs in locomotive modes beyond regular level walking. For example, powered prosthetic joints may enable amputees to climb stairs in a more natural way (Lawson et al., 2013). However, to support such multi-functional operation in amputee users’ daily life, reliable identification of users’ motion intent (as represented by the desired mode of locomotion) is indispensable, as each locomotive mode requires a specifically designed motion control strategy to fit its unique dynamic characteristics. Furthermore, an even greater challenge is the timely recognition of the amputee user’s intent of locomotive mode transition. When a prosthesis motion controller transitions from the current mode of operation to a new mode (e.g., walking to stair climbing), such transition needs to occur on a timely basis (with minimal time delay) to avoid disrupting the amputee user’s overall gait control. Considering the weak gait and stability control capability of lower-limb amputees as well as the increased risk of fall during such transitional movements, the ability of recognizing mode transitions and taking the corresponding control actions is critical for the amputees’ mobility and safety in daily living.

Motivated by the importance of the topic, intent recognition for prosthesis control has been investigated by numerous investigators in the area. Two types of sensor signals were used as the major sources of information. The first is the muscle activation signals acquired through surface electromyography (sEMG). For example, Huang et al. developed phase-dependent sEMG pattern recognition methods using linear discriminant analysis (LDA) and artificial neural network (ANN) classification techniques to recognize multiple modes of locomotion, including standing, level walking, and stair ascent/descent (Huang et al., 2009; Huang et al., 2011); recently, Zhang et al. developed a dynamic adaptive neural network algorithm for the multi-feature fusion-based processing of sEMG signals (Zhang et al., 2022). With the sEMG serving as a noninvasive interface to the user’s nervous system, the acquired sEMG signals may directly reflect his/her intent for the desired joint motion. However, sEMG also suffers from multiple issues such as low reliability and weak signals susceptible to noise and motion artifacts, affecting its practical use in amputees’ daily life. The other type of sensor signals is the signals from mechanical sensors, most of which are embedded in the prosthesis itself (joint angles/angular velocities, accelerations/angular velocities measured through inertia measurement units, ground reaction forces, etc.). For example, Varol et al. developed a Gaussian Mixture Model (GMM)-based supervisory controller of powered lower-limb prostheses to infer users’ intended motion modes (stand, sit, or walk) based on the signals from the prosthesis-embedded joint motion and interaction force sensors (Varol et al., 2010). More recently, Su et al. developed a convolutional neural network (CNN)-based method to recognize human motion intent utilizing the signals from the inertia measurement units (IMUs) mounted on the healthy legs of lower-limb amputees (Su et al., 2019); Cheng et al. developed a biomechanically intuitive activity recognition approach using the signals from a thigh-mounted IMU and a force-sensing resistor as the input (Cheng et al., 2021). Additionally, fusion of the sEMG and mechanical sensor signals has also been investigated to improve the intent recognition performance (Huang et al., 2011; Young et al., 2013a; Young et al., 2013b).

Despite the large body of research work dedicated to the topic, reliable real-time recognition of user motion intent, especially on the desired locomotive transition, still remains a challenging issue that affects robotic prostheses’ practical use in amputees’ daily life, as the majority of existing approaches are only capable of recognizing the current (on-going) locomotive mode. Further, the heavy computation load associated with many intent recognition approaches also hampers their implementation in prosthesis control systems due to the limited computational power of the onboard microcontrollers. To overcome these challenges, the authors present a new lightweight intent recognizer to detect the user’s desired locomotive mode transition early in the swing phase, such that a robotic lower-limb prosthesis may assist the amputee user to complete the power-demanding portion of the gait cycle with its powered joint actions. Such early and timely recognition of the locomotive transition may form an important building block for a future versatile (multi-modal) prosthesis control system to facilitate robotic prostheses’ use in amputees’ daily life.

As the basis of the intent recognizer development, the authors completed a multi-modal gait data collection study, including a variety of locomotive modes and mode transitions (detailed in the subsequent section). To facilitate the intent recognizer’s implementation in real-time prosthesis control, the proposed intent recognizer only involves the signals from common mechanical sensors, including joints angle and inertial measurement data. Utilizing these sensor signals, the authors developed a multidimensional dynamic time warping (mDTW) method to provide timely detection of possible walking-to-stair ascent/descent transitions in the swing phase (Section 2), such that the robotic prosthetic joints may assist the user to complete the potentially power-demanding actions during the subsequent stance phase (e.g., lifting of the body center of mass during stair ascent). The mDTW method also provides an additional advantage of facilitating personalized and continuous adaptation through supplemental template generation, which may be especially useful for amputee prosthesis users with highly diverse and evolving gait patterns.

## 2 Methodologies

### 2.1 Gait data collection study

**FIGURE 1:**
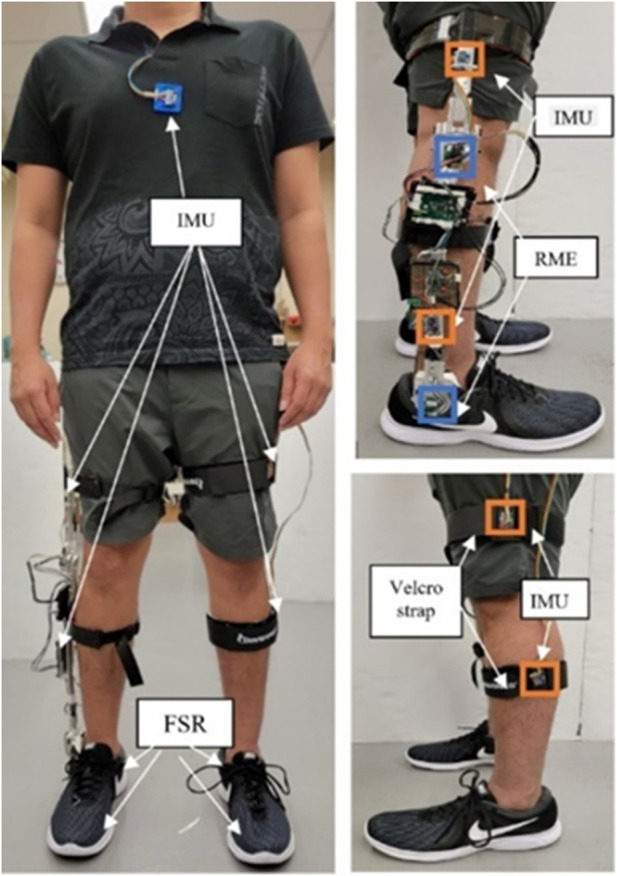
Prototype of the measurement exoskeleton.

To support the development of the intent recognizer, a study was conducted to collect the related gait data. In this multi-modal gait data collection study, the sensors were selected primarily based on the availability in robotic lower-limb prostheses. The majority of sensors used in this study were those embedded in a lower-limb exoskeleton, which are able to measure limb movement with high accuracy and reliability (Haque et al., 2019; Haque et al., 2021). Considering the fact that most lower-limb amputees are unilateral, a single exoskeleton was attached to each participant to measure the knee and ankle joint movement (using rotary magnetic encoders), the shank and thigh 3D movement (using two inertia measurement units (IMUs)), and the foot plantar pressure (using two force-sensing resistors (FSRs) embedded in the shoe). These sensor signals are expected to be available from a robotic lower-limb prosthesis. Further, wearable sensors were attached to other parts of the human body to provide additional gait information, including two IMUs attached to the contralateral leg (shank and thigh), an additional IMU attached to the chest, and two FSRs embedded in the shoe of the contralateral foot (on the heel and first metatarsal head to facilitate the detection of important gait events such as heel strike and toe-off). Details of the sensor placement are show in Figure 1. Nine subjects with no physical and cognitive abnormalities (anthropometric data shown in Table 1) participated in the study. Note that the study were conducted on healthy subjects for two main reasons: 1) the target users of the proposed mDTW method (individuals with amputation fitted with future robotic prostheses) may be able to walk like healthy individuals, and thus the corresponding gait data would be similar to those of healthy individuals as well; and 2) it is difficult to recruit participants from the target user population, as the use of robotic protheses is still very limited (note that the walking gait of amputees fitted with traditional passive prostheses is significantly different from that of amputees fitted with robotic prostheses). The study was approved by the Institutional Review Board (IRB) at the University of Alabama. After the exoskeleton and the wearable sensor were attached, each subject was asked to walk freely for 3–5 min to get comfortable with the setup. Subsequently, the subject performed the following locomotives activities: a) walk on treadmill at self-selected slow, moderate, and fast speeds (each speed for 30 s), b) perform a total of four sequences of motion activities comprising all three motion states, such as level ground walking, walking to stair climb transition, and walking to stair descend transition.

**TABLE 1:** Anthropometric Data of the participants.

Subject	Gender	Age (year)	Weight (kg)	Height (cm)
1	Male	26	84	177.8
2	Male	30	64	175.25
3	Male	26	78	172.72
4	Male	30	55	169
5	Male	26	72	1.75.25
6	Male	27	84	167.64
7	Male	32	63.5	167.64
8	Male	25	61	162.56
9	Male	25	76	170.18

The activities within the four sequences were organized in different orders to avoid bias in the data collection. The experiments were conducted using two staircases (one staircase with a left hand turn and the other with right-hand turn) connected with a long straight hallway. Activity sequence started from stair descent in one sequence and stair ascent in the other one for each staircase selected. As such, a total of four activity sequences were tested based on the starting points of the sequences. Similarly, the four sequences ended at four different stopping points. The walking speeds within the sequence were randomized among three self-selected speeds (fast, normal and slow speed). The participants were free to take rest whenever necessary. The entire experiment was videotaped with a handheld camera. Before starting the data collection, the camera and the exoskeleton system were time synchronized. A desktop computer was used to send timestamps to the sensor system while the camera used its own application to synchronize the time. The activities to be recognized and the corresponding durations are listed in Table 2.

**TABLE 2:** Activities and the corresponding durations.

Activities	Time (sec)
Walking	4032.38
Walking to stair climbing	25.56
Walking to stair descending	23.19

On average, 40 min of data were recorded per subject. The data contains values of the accelerometer and gyroscope for x, y, and *z*-axes from five IMUs, the joint position of knee and ankle, as well as the heel and the ball pressure from the FSRs under the left/right feet. In addition to the signals from the sensors, we also extracted the thigh angle (with respect to the vertical direction) from the corresponding IMU signals, considering the fact that the thigh movement directly reflects a person’s intended motion (e.g., raising the thigh higher in stair climbing). Specifically, complementary filter and Kalman filter were used to extract the thigh movement. First, accelerometer data was used to calculate the angles (roll and pitch). Subsequently, gyroscope data were integrated to get pitch and roll rates. Following this, the accelerometer and gyroscope data were combined to obtain a filtered estimate. Next, the Kalman Filter was initialized by defining state variables, matrices, and initial conditions. After that gyroscope data were used to predict the next state. Following the prediction, accelerometer data were incorporated to correct the predicted state. Ultimately, angles were extracted from the corrected state.

The recorded signals were then processed using a MATLAB script for noise removal. A second-order low-pass Butterworth filter with 15 Hz cutoff frequency was applied to individual sensor signals. Afterward, the signals were normalized using MATLAB “normalize” function.

### 2.2 Data labeling and augmentation

Human locomotion is cyclic in nature. In data processing, the data sequences were segmented into gait cycles by monitoring the shank IMU accelerometer *z*-axis data and the heel pressure data. Note that, different from the traditional method of starting each gait cycle from ground contact (i.e., stance phase first), the segmented gait cycles in this work start from the event of toe-off (i.e., swing phase first). Such method of segmentation enables the proposed intent recognizer to recognize the desired mode (or mode transition) early in the swing phase, which, in turn, enables the prosthesis motion controller to regulate the actuator power output to assist the amputee user to complete the often power-demanding stance-phase motion. Subsequently, the cycles were manually labeled using MATLAB signal labeling toolbox using the video as the reference. Related to the intent recognition in this work, three types of cycles were utilized, including level walking (LW), level walking to stair climbing (LW-SC) transition, and level walking to stair descending (LW-SD) transition. In the data set, the number of LW cycles was significantly larger than the transitional motion cycles. To address this imbalanced dataset issue, all transitional motion states cycles were augmented by (a) scaling (96%, 98%, 102%, and 104% of the original amplitude) (b) resampling, and (c) white Gaussian noise augmentation (SNR 30 dB, 35 dB, 40 dB, and 45 dB) (Wen et al., 2021). The post-augmentation dataset contained 30,461 LW cycles, 2392 LW-SC cycles, and 2184 LW-SD cycles.

### 2.3 Dynamic time warping

**FIGURE 2:**
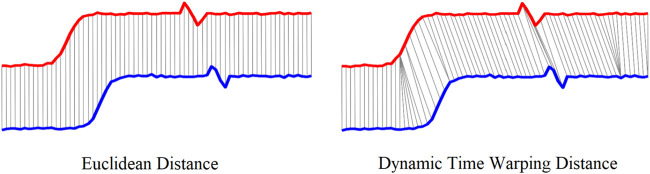
Euclidean distance vs. Dynamic time warping distance.

Leveraging the cyclic nature of human locomotion, the proposed intent recognition algorithm was developed by comparing the real-time sensor signals with the known patterns of locomotion based on the progression in a gait cycle. The comparison was conducted with the method of Dynamic Time Warping (DTW) (Liberman, 1983), which was developed to compute the optimal match between two given signal sequences. The DTW is very efficient in the time-series similarity measurement even if the two time-series are not aligned in the time axis despite being very similar in shape. While Euclidean distance assumes the *n*th point in one sequence is aligned with the *n*th point in the other, DTW alignment allows a more intuitive distance measure to be calculated, as shown in Figure 2. The DTW distance is expected to be much smaller compared with the Euclidean distance after optimally matching the signal sequences. The equations below outline the core steps of Dynamic Time Warping, providing a mathematical framework for aligning sequences (Jang et al., 2017; Weng et al., 2023).

In the proposed DTW-based intent recognition algorithm, we express any movement gait cycle from a continuous movement sequence and a template cycle as two time series *X* and *Y*.

$$X=\left(x_{1},x_{2},\ldots \ldots ,x_{m}\right)$$ (1)

$$Y=\left(y_{1},y_{2},\ldots \ldots ,y_{n}\right)$$ (2)

where *m* is the length of *X* and *n* is the length of *Y*. A distance matrix *D* in the size of (*m×n*) is formulated using the single-point Euclidean distance between *x*_*i*_ and *y*_*j*_ of the sequences *X* and *Y*:

$$D={\left[\begin{array}{ccc} \begin{array}{cc} D_{1,1} & D_{1,2}\\ D_{2,1} & D_{2,2} \end{array} & \begin{array}{c} \cdots \\ \ldots \end{array} & \begin{array}{c} D_{1,n}\\ D_{2,n} \end{array}\\ \begin{array}{cc} \vdots & \vdots \end{array} & D_{i,j} & \vdots \\ \begin{array}{cc} D_{m,1} & D_{m,2} \end{array} & \cdots & D_{m,n} \end{array}\right]}_{m\times n}$$ (3)

The cumulative distance matrix *C* is calculated where each element *C* (*i,j*) represents the cumulative distance from the starting point (1, 1) to cell (*i*, *j*) using dynamic programming:

$$C\left(i,j\right)=D\left(i,j\right)+min\left\{\left(C\left(i-1,j\right),C\left(i,j-1\right),C\left(i-1,j-1\right)\right\}\right\}$$ (4)

The initial conditions are *C* (1,1) = *D* (1,1), *C* (*i*,1) = *D* (*i*,1)+*C* (*i*−1,1), and *C* (1,*j*) = *D* (1,*j*)+*C* (1,*j*−1). The optimal warping path is calculated through the cumulative distance matrix, using backtracking to trace the path with the minimum total distance. This path represents the alignment between the two sequences. The total distance along the optimal warping path is the sum of the distances between the aligned elements.

As described above, the DTW algorithm calculates the warping path which gives the lowest distance/cost measure between *X* and *Y*. The measured cost/distance should be low if *X* and *Y* are alike and high if they are dissimilar. The multidimensional Dynamic Time Warping (mDTW) algorithm is an extension of the regular DTW algorithm that takes all dimensions into account when finding the optimal match between two series. As multiple sensor signals are available to support the intent recognition, the mDTW was adopted to make full use of the rich information embedded in the sensor signals. Further, based on the key requirement of identifying the possible locomotive mode transition early in the swing phase, the cumulated real-time sensor signals from the start of the gait cycle were compared with the corresponding templates with the progression of the gait cycle, which may generate the valuable information of intent recognition performance and its improvement when more sensor information becomes available with the progression in the gait cycle.

### 2.4 Template generation

As described earlier, a total of three locomotive states (mode or mode transitions) were investigated in this study, including a steady state (level walking, LW) and two transitional state (level walking to stair climbing, LW-SC, and level walking to stair descending, LW-SD). Leveraging the cyclic nature of human locomotion, we also segmented the gait data into individual cycles, starting from the event of toe-off (exoskeleton side). Note that such definition was applied to all gait cycles in this work, including the steady-state LW cycle as well as the transitional (LW-SC and LW-SD) cycles.

**FIGURE 3:**
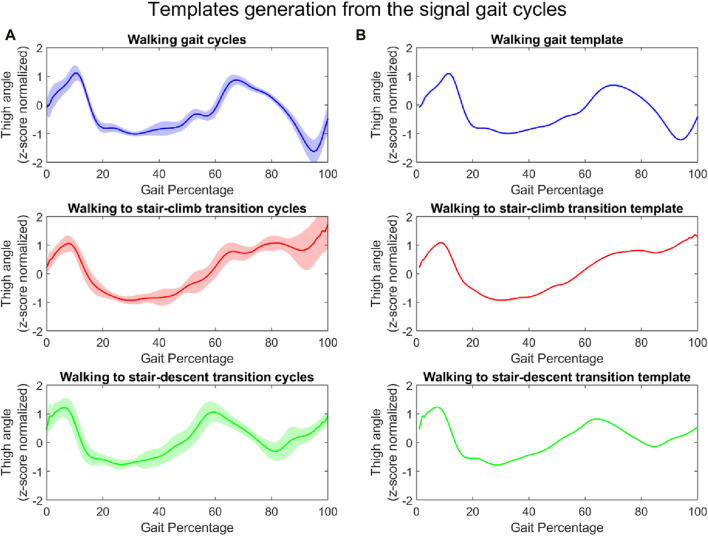
Thigh angle: signal gait cycles of the three locomotion modes (each shaded region represents one standard deviation) **(A)** and their respective templates **(B)** (all normalized).

Utilizing the collected gait data, templates were generated to represent the characteristics of each mode (mode transition). Specifically, the templates were generated by averaging (sample by sample) all gait cycles for the respective mode or mode transition. To address the slight variation of cycle length, the gait cycles were resampled to the average length of the gait cycles using the Matlab ‘resample’ function prior to averaging. The templates were generated for all motion modes and all sensor signals, including the thigh angle, all axes of the gyroscopes and accelerometers in the IMUs, joints angles, and foot pressures. As an example, the templates of the thigh angle for the three locomotion states are shown in Figure 3. Figure 3A shows the signal cycles from the study, while Figure 3B shows the generated templates for the respective locomotion modes.

### 2.5 Classification with multiple templates and leave-one-out validation

**FIGURE 4:**
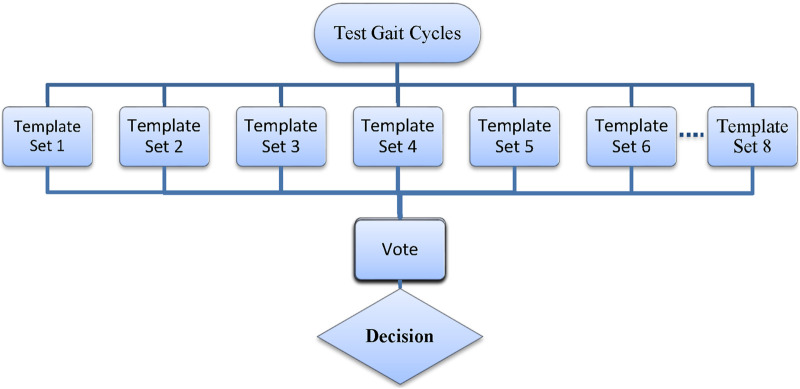
Intent recognition classification model.

With the availability of multiple templates from different test participants (a total of 111 templates for all signals and locomotive states from each participant), the standard mDTW method was augmented with a voting mechanism, which exploits the individual predictions to make the final prediction (Figure 4). The majority voting scheme has been used in a variety of methods, and it can be shown that majority voting improves the probability of correct classification regardless of the type of classifier used (Narasimhamurthy, 2005). Incorporating the voting mechanism is expected to improve the performance of the mDTW intent recognizer by accommodating the variation of human gait patterns among different individuals. When multiple sets of templates were obtained from multiple human subjects, the voting mechanism can be combined with the standard mDTW method to make full use of the available template sets and provide more accurate and responsive intent recognition. To classify an unknown motion cycle, the corresponding sensor signals are compared with the available template sets to compute the similarity scores, generate individual predictions, and finally lead to the final prediction through voting. Specifically, comparison with each template set (associated with each subject) yields an individual prediction (LW, LW-SC, or LW-SD) based on the similarity score within the set; the final prediction is then determined by the collection of individual predictions through voting. In the event of a tie, the final decision will be made through the comparison of average similarity score.

Considering the large number of signals available, a forward selection process was implemented to identify a subset of signals with the most significant contributions to the classification. This was an iterative process, starting from the signal with the best performance of classification when used as the single input to the algorithm. In each iteration, a new signal was added, which best improved the model till an addition of a new variable did not improve the performance of the model.

For the validation of the intent recognition algorithm, the standard leave-one-subject-out method was adapted to this specific application. Specifically, for the performance characterization on each subject, the corresponding template set (i.e., generated by his/her own data) was excluded. Such cross-validation is expected to generate an unbiased evaluation of the intent recognizer while making full use of the gait data available.

## 3 Results

As described in Section 2.5, the forward selection method was utilized to determine the most significant set of signals, along with the optimal number of dimensions, for implementing the mDTW method. The list of sensors signal selected from the forward selection method is tabulated in Table 3. Maximum accuracy was achieved when six sensor signals were used in this method. Table 4 shows the overall accuracy and F1-score of the proposed method by increasing the dimension from one to six using the forward selection method. The table also shows that the accuracy reduces by adding an additional sensor signal other than selected by the forward selection method in the mDTW model; hence only six sensor signals were considered in this model. The last column in Table 4 shows the inference (classification) time for the algorithm executed in Matlab 2023a (DTW function from the Signal Processing Toolbox) on a 3.2 Ghz Intel Core I-9 processor.

**TABLE 3:** List of sensor signals used in mDTW.

Sensor ID	Sensor description
Sig1	Hip angle (Instrumented Side)
Sig2	Hip angle (Un-instrumented Side)
Sig3	Accelerometer-Y (Un-instrumented shank)
Sig4	Gyroscope-Z (Instrumented thigh)
Sig5	Accelerometer-Y (Chest)
Sig6	Accelerometer-Z (Chest)
Sig7 (not selected)	Accelerometer -X (Instrumented thigh)

**TABLE 4:** Performances of the method for different dimensions.

List of sensor signals	Overall accuracy (%)	F1 score	Inference time (msec)
Sig1	68.90	0.6527	0.92
Sig1, Sig2	85.29	0.8399	2.03
Sig1, Sig2, Sig3	90.43	0.8913	2.35
Sig1, Sig2, Sig3, Sig4	92.52	0.9414	2.53
Sig1, Sig2, Sig3, Sig4, Sig5	97.05	0.9544	2.83
Sig1, Sig2, Sig3, Sig4, Sig5, Sig6	99.08	0.9730	3.24
Sig1, Sig2, Sig3, Sig4, Sig5, Sig6, Sig7	95.06	0.9423	3.45

**FIGURE 5:**
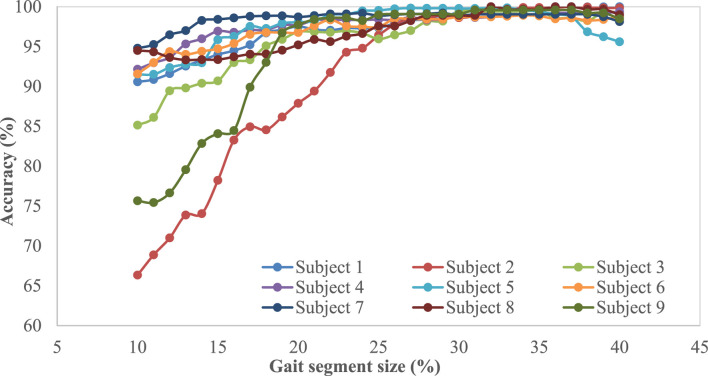
Accuracy vs. gait segment size for different participants.

**FIGURE 6:**
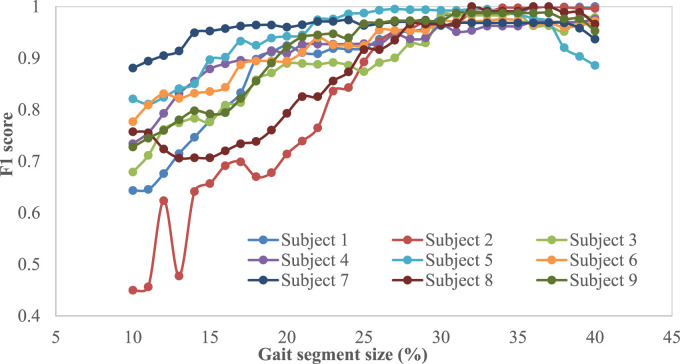
F1-score vs. gait segment size for different participants.

Note that, when implementing the proposed intent recognizer in prosthesis control, the cumulated real-time sensor signal data from the beginning of the gait cycle will be used to compare with templates for locomotive mode recognition, and reliable recognition early in the swing phase is highly desirable. As such, performance of the proposed mDTW algorithm was investigated for the different segment sizes (percentages from the initiation of the gait cycle). Figure 5 and Figure 6 show the performance (accuracy and F1 score, respectively) of the mDTW algorithm with respect to the percentage of the gait cycle. For most subjects, both accuracy and the F1-score increase with the progression in the gait cycle. At 30% gait cycle, the accuracy of classification for all subjects exceeded 98%, suggesting that the mDTW algorithm was able to recognize the potential transitions (LW-SC and LW-SD) well before reaching the stance phase [typically starting at ∼40% of the gait cycle if starting from toe-off (Winter, 2009)].

**FIGURE 7:**
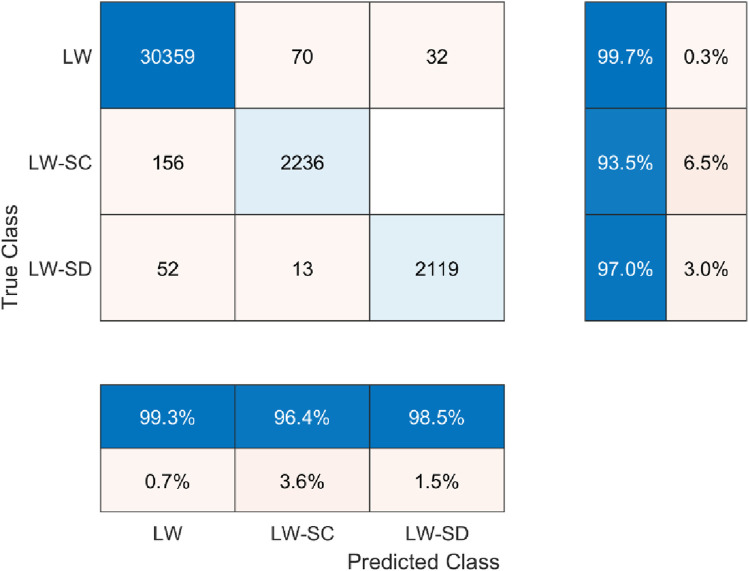
Confusion matrix of the testing for 30% gait segments.

To provide more quantitative performance information of the proposed mDTW algorithm, the cumulative confusion matrix for 30% gait cycle is shown in Figure 7. As can be clearly observed in this figure, the data obtained in the first 30% of the gait cycle enabled the mDTW algorithm to recognize the locomotive mode and mode transitions with high accuracy, providing sufficient time for the lower-level prosthesis motion controller to switch to the correct mode of operation and complete the power-demanding portion (typical stance phase) of the gait cycle.

**FIGURE 8:**
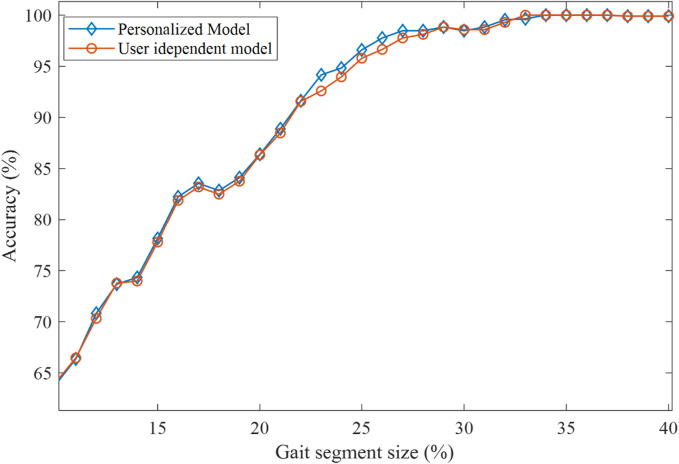
Comparison of classification accuracy between personalized model vs. user independent model.

As the final part of the testing and validation, we investigated the potential of personalization using a subject’s own (personalized) templates. Specifically, half of the subject’s data were used to generate a set of personalized templates, while the other half were used for validation and performance characterization. The performance of this personalized mDTW algorithm (with user-generated templates) was then compared with the performance of the user-independent mDTW described above, with the typical results shown in Figure 8. As can be observed in this figure, the personalized mDTW algorithm was able to improve the recognition performance in a certain range of gait cycle percentage, but the magnitude of improvement was not significant. The reason, presumably, is that the study only involved healthy subjects with similar normal walking gaits, which diminished the performance enhancement provided by the personalization.

## 4 Discussion

Leveraging the data generated by multiple sensors in a multi-modal gait data collection study, we developed an mDTW intent recognizer to detect the locomotive mode or mode transition early in the swing phase. Note that a variety of machine learning methods (models) were developed for lower-limb prosthesis control-oriented user intent recognition. For example, Bhakta et al. (2020) compared the performances of three machine learning algorithms in lower-limb prosthesis control-oriented intent recognition, including linear discriminant analysis (LDA), neural networks (NN), and XGBoost. Based on the results reported in this paper, when leave-one-out validation was conducted, the XGBoost method outperformed the other two methods, with the errors of 10.12% in recognizing steady-state locomotive modes and 15.78% in recognizing mode transitions. Note that, the intent recognition models tested in this paper only recognize which mode (or mode transition) each step belongs to, using the sensor signal data collected in locomotive experiments. In comparison, the mDTW intent recognizer in this paper is capable of detecting the ongoing mode transition during the transitional gait cycle, and thus avoids the typical one-step delay in mode transition recognition. Regarding the comparison of recognition accuracy, our mDTW model was able to provide lower error (<6.5%) than the models tested in Bhakta et al., 2020 when only level walking to stair climbing and stair descending transitions were considered. Finally, the proposed mDTW model features low computational load and fast inference time (Table 4), beneficial for its implementation in the real-time control of robotic lower-limb prostheses.

While 36 collected sensor signals were investigated in this study, all sensors’ signals are not equally useful in classifying the intended motion states. Besides, as the number of signals increases, the computational load required for robotic prosthesis control also increases. Therefore, it is necessary to select the most useful signals while keeping the number of signals to a minimum. The selected signals using the forward selection algorithm are tabulated in Table 3. Table 4 shows a clear upward trend of the accuracy and F1-score by adding more dimensions in the mDTW model. The results shows that the accuracy of the model has been significantly improved (from to 68.90%–99.08%) while six dimensions were used instead of a single dimension. However, the accuracy does not improve beyond these six sensors signals.

Based on the results shown in Figure 6, the accuracy and F1-score show increasing trends with respect to the percentage of progression in the gait cycle (starting from toe-off) irrespective of the participants. A few participants (subject-6, subject-2, and subject-8) show low accuracy and low F1-score at 10% and 20% segment size; however, all participants show more than 98% accuracy and 0.97 F1-score respectively at 30% into the gait cycle. The figure also shows that the accuracy and F1-score do not improve significantly when the progression in the gait cycle exceeds 30%; hence this size could be considered the optimal point of decision for this method. This also suggests that this method can predict the intended modes with high accuracy within the swing phase to provide sufficient time for the prosthesis motion controller to switch modes if necessary.

One of the major advantages of this classification model is that it can operate in a user-independent manner. As described in Section 2.5, this method did not use any templates generated from the participant’s own gait data during the validation process. The user-independent classification allows an intent recognition system to be used in an “off-the-shelf” fashion where a single, generic intent recognition system for a robotic lower limb prosthesis reduces personalized training times, which would otherwise be burdensome to the user and the clinician.

However, the model facilitates the use of personalized templates to further improve the performance. Figure 8 shows the how the classification accuracy changes after introducing personalized templates in the model. The results did not show significant improvement of the overall accuracy by introducing personalized templates for the able-bodied participant. However, considering the diverse and time-varying gait patterns of lower-limb amputees, template personalization may become a useful way to improve the intent recognition performance in the real-world application in the robotic lower-limb prosthesis control. In fact the lack of available templates may be a significant challenge when implementing the mDTW intent recognizer in robotic lower-limb prostheses, as the amputee users of such robotic prostheses may display substantially different gait patterns from healthy individuals. A possible solution, presumably, is to incorporate a data collection session when tuning prosthesis controllers for individual users, such that the mDTW intent recognizer can be personalized through the generation of individual templates.

For the future work, the proposed mDTW intent recognizer is expected to be implemented as the upper-level controller in the real-time control system of future robotic lower-limb prostheses. The algorithm will be executed in cycles, with each cycle initiated at toe-off (i.e., start of the stance phase). With the progression of the gait cycle, template comparison will start at approximately 10% of the gait cycle to allow enough data to be accumulated. Subsequently, template comparison will be continuously conducted to recognize possible gait mode transition. Note that, the proposed mDTW intent recognizer can be easily integrated with the finite-state impedance controller (FSIC), the most widely used lower-limb prosthesis motion control approach, as the FSIC’s controller behavior (mimicking the combination of a virtual spring and a virtual damper) is also gait phase-specific (i.e., controller behavior change triggered by a certain gait event such as toe-off and heel strike) (Sup et al., 2008). Such compatibility is expected to facilitate the mDTW intent recognizer’s future application in the prosthesis control and generate a greater impact in the field. Considering the importance of intent recognition in prosthesis control, a fault detection module (similar to that described in Zhang and Huang, 2015) may be incorporated to detect signal anomaly and improve the algorithm’s reliability through the use of possible recovery mechanisms. Finally, the proposed mDTW method’s functionality may be expanded to recognize other locomotive modes and mode transitions (e.g., stair climbing/descending to walking), and its application may also be expanded to the control of other types of devices assisting the user’s lower-limb motion (e.g., robotic knee exoskeletons).

## 5 Conclusion

In this paper, we developed a new mDTW intent recognition method to recognize the locomotive mode and mode transition in the swing phase of the gait cycle, with the purpose of enabling a robotic prosthesis to assist its user to complete the potentially power-demanding actions during the subsequent stance phase. Through a multi-modal gait data collection study, we obtained the necessary data from multiple mechanical sensors to support the subsequent classifier development. When developing the mDTW algorithm, feature selection was conducted to identify the six most useful sensor signals as the input, and a voting mechanism was used to augment the standard mDTW algorithm to make full use of the gait data obtained on the multiple subjects (through the corresponding templates). Through validation, it was shown that the proposed mDTW algorithm can recognize the locomotive mode or mode transition within 30% progression of a gait cycle with 99.08% accuracy and 0.9730 F1-score. As such, when used in a hierarchical prosthesis control system, such early-swing-phase detection is expected to provide sufficient time for the lower-level motion controller to switch operation mode if necessary before the initiation of the stance phase. Finally, with the algorithm’s low computational load and easiness of personalization through individual template generation, the proposed mDTW intent recognizer may become a basic building block of future prosthesis control systems to facilitate the robotic prostheses’ real-world application among the large amputee population.

## Data Availability

The raw data supporting the conclusion of this article will be made available by the authors, without undue reservation.
